# Plasma Metabolomic Profiling Associates Bicuspid Aortic Valve Disease and Ascending Aortic Dilation with a Decrease in Antioxidant Capacity †

**DOI:** 10.3390/jcm9072215

**Published:** 2020-07-13

**Authors:** Neus Martínez-Micaelo, Carme Ligero, Borja Antequera-González, Alexandra Junza, Oscar Yanes, Josep M. Alegret

**Affiliations:** 1 Grup de Recerca Cardiovascular, Institut d’Investigació Sanitària Pere Virgili (IISPV), Universitat Rovira i Virgili, 43201 Reus, Spain; cligero@grupsagessa.cat (C.L.); borja.antequera@urv.cat (B.A.-G.); 2 Servei de Cardiologia, Hospital Universitari de Sant Joan, Universitat Rovira i Virgili, 43201 Reus, Spain; 3 Metabolomics Platform, Institut d’Investigació Sanitària Pere Virgili (IISPV), Department of Electronic Engineering, Universitat Rovira i Virgili, 43007 Tarragona, Spain; alexandra.junza@urv.cat (A.J.); oscar.yanes@urv.cat (O.Y.); 4 Spanish Biomedical Research Centre in Diabetes and Associated Metabolic Disorders (CIBERDEM), 28029 Madrid, Spain

**Keywords:** bicuspid aortic valve, ascending aortic dilation, metabolomics, alpha-Tocopherol, antioxidant, inflammation

## Abstract

Background: The bicuspid aortic valve (BAV) is the most common cardiac congenital disease and is associated with an increased risk of developing ascending aorta dilation; which can have fatal consequences. Currently; no established risk biomarkers exist to facilitate the diagnosis and prognosis of BAV. Methods: Using an untargeted metabolomic approach; we identified the levels of metabolites in plasma samples and compared them depending on the bicuspid or tricuspid morphology of the aortic valve. Including those patients with ascending aortic dilation and/or aortic stenosis (*n* = 212), we analyzed the role possibly played by alpha-Tocopherol in BAV disease; considering its association with the pathophysiological characteristics of BAV and biomarkers related to inflammation, oxidative stress and endothelial damage, as well as characteristics related to alpha-Tocopherol functionality and metabolism. Results: We found that BAV patients; especially those with ascending aortic dilation; presented lower antioxidant capacity; as determined by decreased plasma levels of alpha-Tocopherol; paraoxonase 1 and high-density lipoprotein (HDL), as well as increased levels of C-reactive protein (CRP; a biomarker of inflammation) and endothelial microparticles (EMPs; an endothelial damage biomarker). By applying random forest analyses; we evaluated the significant screening capacity of alpha-Tocopherol; CRP and EMPs to classify patients depending on the morphology of the aortic valve. Discussion: Our findings support the role of decreased antioxidant capacity; increased inflammation and endothelial damage in the pathogenesis of BAV and the progression of aortic dilation. Moreover; determining the plasma levels of alpha-Tocopherol; CRP and EMPs could improve BAV diagnosis in large populations.

## 1. Introduction

Bicuspid aortic valve (BAV) is the most common cardiac congenital malformation, affecting 1–2% of the population and occurring when two of the three leaflets (or cups) of the aortic valve did not separate during fetal development (or less frequently, when congenitally only two leaflets are presents), leading to a bicuspid configuration, rather than the normal three (tricuspid aortic valve; TAV) [1]. 

As such, BAV patients present a high prevalence of aortic dilation (greater than 50%) which, if untreated, can lead to fatal consequences, such as aortic dissection and/or rupture [2,3]. Bicuspid morphology of the aortic valves causes turbulent blood flow and abnormal hemodynamical patterns in the proximal ascending aorta [4], generating shear stress that impacts the endothelial cells that line the inner surface and leading to dysfunctional aortic endothelium [5,6].

In clinical practice, early detection of BAV is difficult because most of the patients are asymptomatic for a long time and echocardiography is the technique most frequently used to achieve a conclusive diagnosis. Currently, no effective strategies exist to prevent the progression of BAV disease and its related disorders; therefore, the development of new and efficient approaches require a more detailed understanding of the molecular mechanisms for BAV pathogenesis [7]. Echocardiographic measurement of the ascending aortic diameter is the most current method used to monitor aortopathy in BAV patients. Therefore, development of new screening methods and identification of non-invasive biomarkers that could be examined in a high-throughput format could facilitate and improve the prompt diagnosis of BAV. 

Untargeted metabolomic profiling is considered a powerful analytic tool for the identification of disease-related biomarkers and pathways that can be used to develop new diagnostic and treatment methods [8]. Considering our previous publications that describe an association between BAV and the circulating levels of biologically active molecules, including microparticles and microRNAs [9,10,11], in this study, we hypothesized that the complex etiology of the BAV valvulopathy and aortopathy could be associated with a differential circulating profile of metabolites, which could supply an improvement in the diagnostic methods and could also elucidate the molecular mechanisms and functional implications underlying BAV. 

## 2. Methods

### 2.1. Study Participants

In this study, we included patients belonging to a cohort of TAV controls and BAV patients prospectively included and followed-up in our facilities. The participants were informed and provided written consent. BAV was diagnosed when two aortic leaflets were visualized, with or without a raphe, in the parasternal short-axis view of a transthoracic or transesophageal echocardiogram [12] or on a cardiac magnetic resonance image [13]. Explorations were performed or supervised by the same observer (JM.A.). This study was conducted according to the principles of the Declaration of Helsinki and was approved by the Institutional Review Board and Ethics Committee (03-06-19/6proj4 and 114/2020) of our institutions, the *Hospital Universitari de Sant Joan* and the *Institut d’Investigació Sanitària Pere Virgili*.

The design of this study included two evaluations of four independent cohorts of age 18–78 years, including TAV individuals (TAV), BAV patients with no aortic dilation (BAV), patients with BAV and ascending aorta dilation at the time of diagnosis (BAVdil) and patients with TAV and ascending aorta dilation (TAVdil) (*n* = 212). The patient characteristics are described in Table 1. First, we determined the influence of the aortic valve morphology on the plasma metabolome by comparing the circulating metabolite profile between TAV and BAV patients. For this purpose and to exclude the effects of possible confounding factors, we included only those TAV (*n* = 33) and BAV patients (*n* = 62) with no aortic dilation (i.e., patients with a diameter of the ascending aorta less than 37 mm) and with no aortic stenosis (defined as patients with a mean transaortic pressure gradient less than 20 mmHg). However, in the second evaluation and to gain insight into the molecular mechanisms underlying the complexity of BAV pathology and the predictive value of the identified metabolites, we included patients with both morphologies (tricuspid or bicuspid) and aortic dilation (TAVdil; *n* = 35 and BAVdil; *n* = 82), as well as patients with aortic stenosis independent of the morphology of the aortic valve.

Patients diagnosed with Marfan syndrome or diabetes mellitus, or those receiving pharmacologic treatment (including statins, ACE/ARBs and/or β-blockers), were excluded. 

### 2.2. Blood Sampling and Analyses

Blood samples were collected under overnight fasting conditions and were processed within 90 min of collection. The samples were centrifuged at 1500× *g* for 15 min to obtain plasma, which was further centrifuged at 4000× *g* for 10 min to obtain platelet-poor plasma. The plasma was stored at −80 °C in our biological samples bank (Biobanc IISPV–HUSJR) until needed.

Total cholesterol, triglycerides, low-density lipoprotein (LDL) cholesterol, direct high-density lipoprotein (HDL) cholesterol, high-sensitivity C-reactive protein (CRP), lipoprotein (a) (Lpa), apolipoprotein B100 and apolipoprotein A1 were measured using standardized enzymatic and immunoturbidimetric assays (Spinreact and Horiba ABX Montpellier) adapted to a Cobas Mira Plus autoanalyzer (Roche Diagnostics). 

The levels of circulating endothelial microparticles (EMPs) were determined as previously described [9]. Briefly, EMPs were measured using flow cytometry (EPICS-XL; Beckman Coulter) at a low rate and 30 s stop time. The Nano Fluorescent Particle Size Standard Kit (Spherotech) and Flow Count fluorospheres (Beckman Coulter) were used in instrument standardization and as an internal calibrator for microparticle amount calculation, respectively.

Plasma EMPs were defined as particles > 0.1 and <1 μm in size and their endothelial origin was identified based on their affinity to specific cell surface antigens, CD31 and CD42b. Possible contamination with leukocyte-derived microparticles was discarded by measuring <4.5% of microparticles co-expressing CD31^+^CD45^+^. EMPs were measured by trained technicians who were blind to the clinical status of the patients as well as to the results.

### 2.3. Metabolomics Profiling

A biphasic extraction with methanol in 0.1% formic acid and dichloromethane was used to extract metabolites. Methanol in 0.1% formic acid was added to plasma samples followed by vortexing and 3 volumes of dichloromethane and 1 volume of water were added. After a 30-min incubation at 4 °C, samples were centrifuged (15,000 rpm, 15 min at 4 °C) and the organic phase was collected for drying under nitrogen. Pellets were resuspended in methanol:toluene (9:1) for LC/MS analysis. 

Untargeted liquid chromatography/mass spectrometry (LC/MS) analyses were performed using an ultrahigh-performance liquid chromatography (UHPLC) system (1290 Agilent) coupled to a 6550 ESI-QTOF MS (Agilent Technologies) instrument operating in positive (ESI+) electrospray ionization mode. Metabolites were separated by reverse-phase chromatography with an Acquity UPLC C18-RP instrument (ACQUITY UPLC BEH C18 1.7 µM, Waters). Mobile phase A was acetonitrile/water (60:40) (10 mM ammonium formate) and mobile phase B was isopropanol/acetonitrile (90:10) (10 mM ammonium formate). Solvent modifiers were used to enhance ionization and to improve the LC resolution in positive ionization modes. The separation was conducted under the following gradient—0 in 15% of B; 0–2 min 30% of B; 2–2.5 min 48% of B; 2.5–11 min 82% of B; 11–11.5 min 99% of B; 11.5–12 min 99% of B; 12–12.1 min 15% of B; and 12.1–15 min 15% of B. The ESI conditions were applied as follows—gas temperature = 150 °C; drying gas = 13 L min^−1^; nebulizer = 35 psig; fragmentor = 150 V; and skimmer = 65 V. The instrument was set to work over an m/z range of 50–1200 with an acquisition rate of 3 spectra/sec. For compound identification, tandem mass Spectrometry (MS/MS) analyses were performed in targeted mode with a default iso-width (width at half-maximum of the quadrupole mass bandpass used during MS/MS precursor isolation) of 4 m/z. The collision energy was fixed at 20 and 40 V. 

LC/MS data were processed using XCMS [14] software (version 1.34.0) to detect and align mzRT features. XCMS analysis of these data produced a matrix containing the retention time, m/z value and integrated peak area of each feature for each sample. We constrained the initial number of features via the following criteria. Only features above an intensity threshold of 5.000 were retained for further statistical analysis. Quality control samples (QCs) consisting of pooled samples from each condition were injected at the beginning and periodically throughout the worklist. The performance of the LC/MS platform for each mzRT feature detected in the samples was assessed by calculating the relative standard deviation of these features on pooled samples (CVQC) [15]. Statistical analyses were performed using the specmine package [16] in R Bioconductor.

### 2.4. Random Forest Model Construction and Validation

A model was constructed to estimate the capacity to predict or classify patients based on the morphology of the aortic valve. Therefore, the selection of the variables that make the largest contributions to classification was conducted using association based on Spearman’s correlation and linear regression analysis and random forest variable importance measures. The analyses were performed using the randomForest package [17] in R (Bioconductor). We build a model to discriminate patients depending on the morphology of their aortic valve. This model was evaluated including patients with no aortic dilation (BAV and TAV, *n* = 95) or including the four previously described groups (TAV, TAVdil, BAV and BAVdil, *n* = 212). Patients were randomly divided into two subsets—63% of the individuals were used to train the model and 37% of the individuals were used to test the developed models. The training data set was evaluated with a 10-fold cross-validation fold procedure. The statistical parameters for assessing the predictive performance of the random forest classifier were based on the accuracy, sensitivity and specificity. 

### 2.5. Statistical Analysis

Data are expressed as the mean ± standard error mean (SEM). Continuous variables were compared between groups using the Wilcoxon test. Correlations were evaluated using Spearman’s Rho. Statistical analyses were performed using SPSS software (IBM SPSS Statistics, United States, version 22.0) and R (Bioconductor). *P*-values < 0.05 were considered to be significant.

## 3. Results

### 3.1. Circulating Profile of Metabolites Depends on the Morphology of the Aortic Valve

To analyze whether BAV valvulopathy could be related to a differential profile of circulating metabolites, we compared the circulating metabolome of patients with bicuspid or tricuspid morphology of the aortic valve using an untargeted metabolomics approach. In this analysis, we selected patients with homogeneous characteristics, no aortic dilation and with no aortic stenosis to exclude the effects of possible confounding factors. 

**Figure 1:**
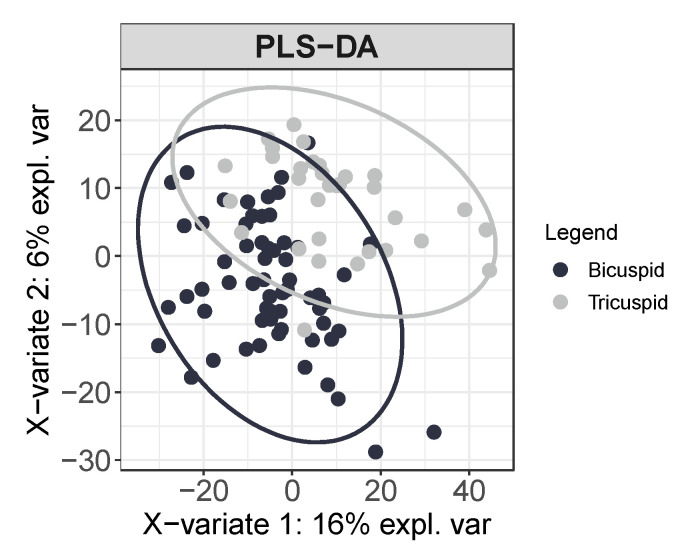
Plasma metabolome differs depending on the morphology of the aortic valve. Multivariate supervised partial-least-squares discrimination analysis PLS-DA of the 2289 total metabolite features detected in plasma classifies between tricuspid aortic valve (TAV) and bicuspid aortic valve (BAV), patients (95% confidence interval ellipses are shown for the TAV and BAV groups). (*n* = 95).

Using this high-throughput approach, we detected 2289 distinct metabolite features in plasma samples. As an exploratory analysis, using unsupervised principal component analysis (PCA), we firstly corroborated the absence of outliers, trends, patterns or clusters in the data prior to statistical analysis. Moreover, using a supervised partial-least-squares discrimination analysis (PLS-DA), we determined that by measuring the metabolomic plasma profile, patients can be classified depending on the bicuspid or tricuspid morphology of their aortic valves (Figure 1). Thus, from the 2289 metabolite features measured, 2 unique metabolites were significantly different between TAV and BAV, namely, alpha-Tocopherol (fdr = 2.17 × 10^−3^) and choline (fdr = 8.44 × 10^−3^) and both metabolites presented lower abundance in the plasma of BAV patients than in that of TAV patients.

### 3.2. Functional Implication of the Identified Metabolites

**Figure 2:**
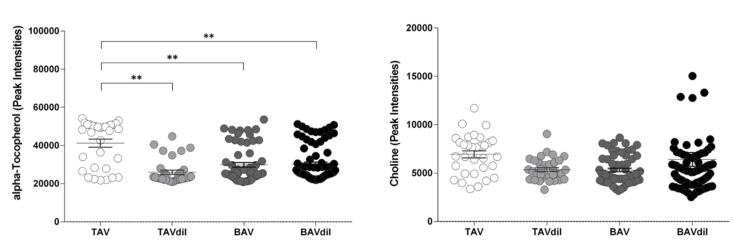
Peak intensities measured for the alpha-Tocopherol and choline levels in TAV and BAV patients. *n* = 202. ** *p*-value < 0.01.

Dilation of the ascending aorta and aortic stenosis are the two most common comorbidities associated with BAV; therefore, to improve the translational character and the clinical applicability of the obtained results, we analyzed whether the circulating levels of alpha-Tocopherol and choline remained significantly different between TAV and BAV patients when we consider those patients with an ascending aortic dilation (TAVdil and BAVdil) and also include those patients with aortic stenosis in the corresponding groups (Figure 2). 

We observed that although the levels of alpha-Tocopherol were higher in TAV individuals than in patients with BAV, BAVdil and TAVdil, no significant changes were observed in the circulating levels of choline. We performed a 3-way ANOVA including the valve morphology, ascending aortic dilation and aortic stenosis as independent variables and alpha-Tocopherol or choline as dependent variables and we found that only the interaction between the valve morphology (BAV vs TAV) and dilation resulted significative (*p*-value = 0.042) for the alpha-Tocopherol levels and no main effects nor interactions resulted significative for the choline levels.

To analyze the relationship between alpha-Tocopherol plasma levels and BAV disease and whether it might play a role in the progression of the disease, we analyzed the association of the circulating levels of alpha-Tocopherol with the pathophysiological characteristics of BAV disease, including echocardiographic and other confounding clinical variables. Using multivariate linear regression analysis (Table 2), we observed that the diameter of the ascending aorta was the only variable independently and negatively associated with the plasma levels of alpha-Tocopherol. 

Using this approach to elucidate the biological implication and the functionality of alpha-Tocopherol in BAV disease, we considered both the cellular and molecular mechanisms that have been associated with BAV, including inflammation, oxidative stress and endothelial damage [6]. Furthermore, we also considered characteristics related to alpha-Tocopherol function and metabolism, including its role as the most important lipid-soluble antioxidant carried by lipoproteins in plasma. Therefore, we first analyzed the effect of aortic valve morphology and ascending aortic dilation on the plasma levels of parameters associated with lipid and lipoproteins, inflammation, oxidative stress and endothelial damage.

We determined that the bicuspid morphology of the aortic valve and dilation of the ascending aorta were related to higher levels of endothelial microparticles (EMPs) and C-reactive protein (CRP), which are biomarkers related to endothelial damage and inflammation, respectively but these patients also had lower levels of apolipoprotein A1 (ApoA1), the major HDL protein (Table 3). 

Using an analysis of covariance (ANCOVA) we found that the levels of alpha-Tocopherol remained significantly different between groups (*p*-value < 0.01) while considering the effect of ApoA1 (*p*-value = 0.423) or HDL (*p*-value = 0.773). 

Furthermore, using Spearman’s correlation coefficient, we found that plasma levels of alpha-Tocopherol were negatively associated with the diameter of the ascending aorta, the aortic root and the age. The plasma levels of alpha-Tocopherol were also associated with circulating concentrations of ApoA1 and albumin, the two carriers of alpha-Tocopherol in plasma and paraoxonase 1, an enzyme with antioxidant properties transported in HDL (Table 4).

### 3.3. Prediction of the Morphology of the Aortic Valve or the Dilation of the Ascending Aorta in BAV Patients

**Figure 3:**
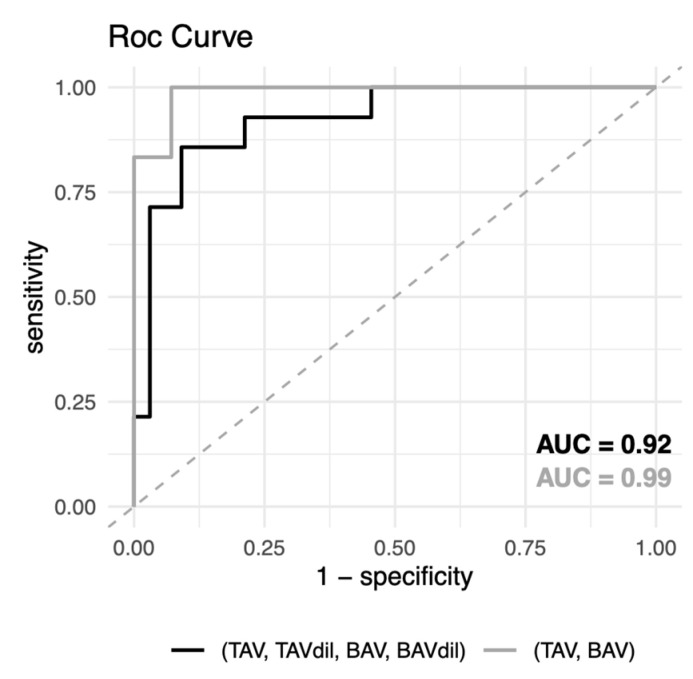
ROC curves of cross-validated random forest classification of test data.

The results obtained in the previous analyses enable us to consider the role of plasma biomarkers based on its functional implication in aortic valve dysfunction and also as predictive circulating signatures of aortic valve morphology. Thus, we evaluated the predictive capacity of the differential metabolomic signature in plasma together with the variables that make the largest contributions to classification. To this end, we used the random forest algorithm to construct a model able to discriminate patients depending on the morphology of the aortic valve (Table 5 and Figure 3). The model was constructed using two approaches, one including all patients independent of the morphology of the aortic valve and the dilation of the ascending aorta and the other excluding those patients with ascending aortic dilation. 

We determined that the alpha-Tocopherol, EMPs and CRP circulating levels were variables able to discriminate patients based on their valve morphologies. Thus, the model constructed using these variables presented an accuracy of 0.89, a test sensitivity of 0.91, a specificity of 0.86 and a *p*-value (accuracy > no information rate) = 3.9 × 10^−^ when we do not exclude patients depending on aortic diameter. However, in the case in which only TAV and BAV patients with no ascending aortic dilation were included in the model construction, the test sensitivity and specificity values were 0.95 and 1.00, respectively and the *p*-value (accuracy > no information rate) = 7.6 × 10^−3^.

## 4. Discussion

Using an untargeted metabolomics approach, we have identified significant associations between the plasma levels of alpha-Tocopherol and the morphology of the aortic valve and diameter of the ascending aorta. Moreover, we have determined that BAV patients, especially those with ascending aortic dilation, are characterized by increased levels of circulating biomarkers of inflammation, such as CRP, endothelial damage by measuring EMPs and decreased antioxidant capacity due to the lower circulating levels of alpha-Tocopherol, as well as its main carrier in plasma, the HDL lipoprotein, measured as ApoA1. Finally, we have evaluated how the circulating levels of alpha-Tocopherol, together with CRP and EMPs, are significant predictors of the aortic valve morphology, thereby identifying alpha-Tocopherol as the most important classification variable.

The pathogenesis of ascending aortic dilation in BAV is not completely understood. Two main non-mutually exclusive theories exist associating BAV aortopathy with genetic or hemodynamic causes. Focusing on hemodynamic causes, the bicuspid morphology of the aortic valve causes an abnormal biochemical environment, a consequence mainly of the non-physiological hemodynamic impact on the ascending aorta. This morphology-associated helical flow alteration that propagates flow eccentricity leads to increased wall shear stress on the endothelium and progressive dilation of the ascending aorta [18,19]. Although BAV disease is asymptomatic initially, the lack of an early diagnosis can lead to identifying the case in advanced stages or after an event. Therefore, in clinical practice, the existing approaches to achieving a conclusive diagnosis and to monitoring aortopathy in BAV patients are based on images obtained by transthoracic echocardiography [20]. A clear need exists for novel diagnostic and molecular tools to improve BAV screening, disease stratification and monitoring response to therapeutic interventions [21]. 

Untargeted metabolomics is a powerful tool that can supply a high-resolution snapshot of the complete set of metabolites, leading to the identification of specific metabolic signatures that can determine any perturbation of single or multiple metabolites and the related biochemical pathways [22], thereby offering new insights into the molecular mechanisms underlying complex diseases. Therefore, despite our untargeted metabolomics approach that identified the alpha-Tocopherol and the choline, only alpha-Tocopherol levels remained significantly different when we included patients with dilated ascending aorta and aortic stenosis, the two common complications related to BAV progression, in the analysis. Alpha-Tocopherol, as the primary form of the vitamin E, is a fat-soluble antioxidant with the capacity to neutralize endogenous free radicals and is carried in the blood primarily through HDL lipoproteins [23] but would also be bound to albumin [24]. Supporting this observation, we found a significant positive correlation between plasma levels of alpha-Tocopherol and ApoA1, albumin and paraoxonase 1, an enzyme transported by HDL with antioxidant and anti-atherogenic properties [25]. Interestingly, in addition to its antioxidant capacity, alpha-Tocopherol also acts as a regulator of the expression of genes involved in lipid metabolism and inflammation [26]. The plasma levels of alpha-Tocopherol have not been previously related to BAV pathology but nevertheless, a previous study Wang et al. [27] identified choline as one of the different metabolites in the serum of BAV patients before aortic valve replacement surgery compared with healthy individuals. However, the patients included in that study presented severe conditions (aortic valve dysfunction and/or aortic dilatation) leading to surgery indication; thus, those conditions together with BAV could affect the circulating metabolome profile. Choline is an essential nutrient for human health, being the liver the central organ responsible for its metabolization. Choline is involved in several physiological functions, phosphorylated, choline acts as the substrate for the synthesis of phosphatidylcholine, a key component of eukaryotic cellular membranes when its oxidized, choline participates as a methyl-donor in the methylation processes and in the case to be acetylated, choline generates acetylcholine, an important neurotransmitter [28,29]. Further, choline also participates in the solubilization of bile salts and is required for the generation of VLDL lipoproteins [30]. Choline has cardioprotective effects by downregulating inflammation, restoring endothelium structure and by inhibiting the generation of reactive oxygen species [28,31].

The results presented in this study showed that circulating levels of alpha-Tocopherol were higher in TAV individuals than in patients with BAV but also in those patients with dilated ascending aorta, that is, including TAVdil and BAVdil patients. Therefore, we speculated that the decrease in the measured levels of alpha-Tocopherol could occur because of cellular and molecular events related to pathogenesis of BAV and vascular wall remodeling associated with dilation of the ascending aorta, rather than as a consequence of the bicuspid morphology of the aortic valve itself. BAV patients normally present increased, even not clinically significant, diameters of the ascending aorta than healthy TAV ones (32.56 ± 0.4 and 30.81 ± 0.6 *p* = 0.003, respectively; Table 1) and according to reported in [32]. However, histological differences exist in the dilated ascending aorta between BAV and TAV patients and several studies have determined that the inherent defects of the aortas in BAV patients lead to altered wall mechanical properties, which might contribute to the progressive dilation of the ascending aorta [33,34].

Using the alpha-Tocopherol levels together with the variables associated with the bicuspid morphology of the aortic valve, that is, the levels of EMPs and CRP, we constructed a model that significantly classified individuals depending on the morphology of the aortic valve. Interestingly, alpha-Tocopherol was identified as the most important variable. Therefore, measuring the levels of alpha-Tocopherol together with the parameters associated with inflammation and endothelial damage might be useful as a more feasible alternative for BAV screening and could be complementary to echocardiogram examination in large populations, as well as being useful in risk stratification of patients susceptible to developing ascending aortic dilation. 

However, considering that the alpha-Tocopherol is functionally related to lipid and lipoprotein metabolism, we must consider whether the endothelial damage and pro-inflammatory state observed in BAV patients, especially in BAV patients with ascending aorta dilation, could be a consequence not only of hemodynamic forces and shear stress due to the bicuspid morphology of the aortic valve but also of an alteration to lipid and lipoprotein metabolism. In support of this hypothesis, selected authors have suggested that several molecular mechanisms that promote atherosclerosis are also present in BAV and that these alterations could underlie the progression of aortic stenosis and dilation [35]. The results reported in this study supported the hypothesis that BAV and dilation of the ascending aorta are associated with increased endothelial damage and inflammation. However, although the results presented in this study support the hypothesis that the lipid and lipoprotein metabolism might play an important role in BAV disease, we cannot establish causality. 

Accordingly, additional studies are necessary to further evaluate whether different lipoproteins profiles in plasma affect the endothelium in BAV patients depending on susceptibility to developing ascending aorta dilation. Additionally, further research is warranted to determine whether alpha-Tocopherol administration might influence the progression of BAV pathology.

## Figures and Tables

**Table 1:** Clinical and echocardiographic characteristics of patients included in this study.

	TAV	BAV	TAVdil	BAVdil	*p*-Value
n	33	62	35	82	
Age (years)	47 ± 2	41 ± 2	63 ± 2	52 ± 2	3.9 × 10^−^^11^ **, b,c,d
Sex (male/female)	(25/8)	(45/17)	(22/13)	(58/24)	0.670
Body weight (kg)	71.32 ± 2.4	71.00 ± 2.0	80.50 ± 11.5	76.52 ± 1.8	0.183
Severe aortic stenosis (mean gradient ≥ 40 mm Hg)	0 (0%)	3 (5%)	0 (0%)	19 (23%)	2.1 × 10^−5^ **
Aortic valve gradient (mean, mm Hg)	3.57 ± 1.0	12.04 ± 1.8	2.85 ± 0.8	24.75 ± 2.6	5.5 × 10^−^^12^ **, b,d
Left ventricle diastolic diameter (mm)	50.22 ± 0.9	52.20 ± 0.8	53.48 ± 1.0	53.79 ± 1.0	0.100
Left ventricle systolic diameter (mm)	30.35 ± 1.2	32.56 ± 0.8	30.01 ± 0.9	33.36 ± 0.9	0.175
Left ventricular ejection fraction (%)	71.92 ± 1.9	70.72 ± 1.2	70.86 ± 1.8	69.18 ± 1.1	0.568
Aortic root diameter (mm)	33.06 ± 0.8	33.93 ± 0.8	41.94 ± 1.2	39.32 ± 0.6	2.4 × 10^−^^13^ **, b,c
Ascending aorta diameter (mm)	30.81 ± 0.6	32.56 ± 0.4	44.88 ± 0.7	44.36 ± 0.6	5.5 × 10^−^^47^ **, b,c

*TAV* tricuspid aortic valve; *BAV* bicuspid aortic valve patients; *BAVdil* patients with bicuspid aortic valve and aortic dilation * Significant values (Overall *p* < 0.01); a, *p* < 0.05 TAV vs. BAV; b, *p* < 0.05 BAV vs. BAVdil; c, *p* < 0.05 TAV vs. TAVdil; d, *p* < 0.05 BAVdil vs. TAVdil. ** *p*-value < 0.01.

**Table 2:** Multivariate linear analysis of variables related to the plasma levels of alpha-Tocopherol.

	Coefficient	Std-Coefficient	*p*-Value
	alpha-Tocopherol		
Age (years)	−47.99 (−157.20 to 61.31)	−0.75	0.388
Aortic root (mm)	120.60 (−207.20 to 448.39)	0.75	0.469
Ascending aorta (mm)	−370.60 (−643.81 to −97.39)	−2.68	8.13 × 10^−3^ **
Mean transaortic pressure gradient (mm Hg)	25.91 (−58.78 to 110.61)	0.046	0.547
Left ventricle diastolic diameter (mm)	29.72 (−316.57 to 376.01)	0.021	0.169
Left ventricle systolic diameter (mm)	133.85 (−418.85 to 686.54)	0.071	0.633
Left ventricular ejection fraction (%)	165.13 (−76.42 to 406.68)	0.157	0.179

** *p*-value < 0.01.

**Table 3:** Effect of aortic valve morphology and ascending aortic dilation on the plasma levels of parameters associated with lipid metabolism, inflammation, oxidative stress and endothelial damage.

	TAV	BAV	TAVdil	BAVdil	*p*-Value
Total cholesterol (mmol/L)	5.61 ± 0.3 (5.04–6.18)	4.77 ± 0.2 (4.43–5.01)	5.20 ± 0.3 (4.60–5.79)	5.07 ± 0.1 (4.79–5.33)	0.171
Triglycerides (mmol/L)	1.36 ± 0.2 (0.96–1.76)	0.97 ± 0.7 (0.82–1.11)	1.48 ± 0.2 (1.13–1.84)	1.33 ± 0.1 (1.06–1.60)	0.139
LDL (mmol/L)	3.45 ± 0.2 (3.00–3.90)	2.87 ± 0.1 (2.58–3.15)	3.26 ± 0.2 (2.80–3.72)	2.97 ± 0.1 (2.74–3.20)	0.172
HDL (mmol/L)	1.55 ± 0.1 (1.32–1.77)	1.47 ± 0.5 (1.37–1.57)	1.26 ± 0.1 (1.14–1.39)	1.49 ± 0.5 (1.39–1.59)	0.049 *
ApoA1 (mg/dL)	160.67 ± 7.3 (144.67–176.66)	133.47 ± 4.4 (124.60–142.35)	139.53 ± 4.5 (130.02–149.03)	140.61 ± 3.7 (133.22–148.00)	0.007 ** a, e
ApoB100 (mg/dL)	118.50 ± 6.9 (103.27–133.73)	88.67 ± 4.1 (80.38–96.95)	112.88 ± 6.2 (99.65–126.12)	99.22 ± 3.7 (91.79–106.64)	0.005 ** a, c
C-reactive protein (mg/dL)	1.06 ± 0.3 (0.38–1.72)	1.71 ± 0.3 (1.14–2.29)	1.84 ± 0.5 (0.86–2.82)	2.42 ± 0.4 (1.70–3.13)	0.046 * e
Paraoxonase 1 (ng/mL)	85.23 ± 6.5 (70.94–99.94)	94.61 ± 8.8 (76.67–112.55)	78.12 ± 8.0 (61.25–94.99)	87.12 ± 7.1 (72.86–101.37)	0.647
Endothelial microparticles (log part/μL)	2.49 ± 0.4 (1.55–3.43)	3.68 ± 0.3 (3.05–4.27)	3.24 ± 0.3 (2.55–3.92)	3.78 ± 0.2 (3.35–4.20)	0.020 * e
Albumin (g/dL)	4.64 ± 0.3 (4.55–4.73)	4.53 ± 0.1 (4.53–4.65)	4.39 ± 0.1 (4.27–4.52)	4.52 ± 0.1 (4.46–4.58)	0.010 * c

* Significant values (Overall *p* < 0.05), ** Significant values (Overall *p* < 0.01), a, *p* < 0.05 TAV vs. BAV; b, *p* < 0.05 BAV vs. BAVdil; c, *p* < 0.05 TAV vs. TAVdil; d, *p* < 0.05 BAVdil vs. TAVdil; e, *p* < 0.05 TAV vs. BAVdil.

**Table 4:** Linear relationship between the plasma levels of the identified metabolites and the parameters associated with lipid metabolism, inflammation, oxidative stress and endothelial damage.

	Alpha-Tocopherol	
	R	*p*-Value
Aortic root (mm)	−0.172	0.012 *
Ascending aorta (mm)	−0.271	7.20 × 10^−5^ **
Mean transaortic pressure gradient (mm Hg)	−0.083	0.230
Age (y)	−0.179	0.009 **
Lipid Metabolism		
Total cholesterol (mmol/L)	0.052	0.514
Triglycerides (mmol/L)	0.023	0.770
ApoA1 (mg/dL)	0.209	0.008 **
ApoB100 (mg/dL)	0.082	0.305
Inflammation		
C-reactive protein (mg/dL)	0.042	0.607
Oxidative stress		
Paraoxonase 1	0.195	0.030 *
Endothelial damage		
Endothelial microparticles (log part/μL)	0.097	0.263
Liver damage		
Albumin (g/dL)	0.248	0.002 **

* *p*-value < 0.05; ** *p*-value < 0.01.

**Table 5:** Evaluation of multivariable biomarker models including alpha-Tocopherol, endothelial microparticles (EMPs) and C-reactive protein (CRP) for the prediction of aortic valve morphology.

*p*-Value	OOB Error	Accuracy (95% CI)	Sensitivity	Specificity	AUC
**TAV + TAVdil + BAV + BAVdil**					
3.9 × 10^−3^ **	21.7%	0.89 (0.77–0.96)	0.91	0.86	0.92
**TAV + BAV**					
7.6 × 10^−3^ **	17.5%	0.95 (0.75–0.99)	0.93	1.00	0.99

** *p*-value < 0.01.

## Limitations

We excluded from the analysis patients with diabetes mellitus and those receiving certain pharmacological treatments such as statins, ACE inhibitors/ARBs and beta-blockers, to minimize the possibility that the excluded variables could influence the identification of related metabolites with the BAV. Subsequent studies carried out in a large population including the excluded variables should validate the generalization of our results.

## Abbreviations

Tricuspid aortic valve	TAV
Tricuspid aortic valve with ascending aortic dilation	TAVdil
Bicuspid aortic valve	BAV
Bicuspid aortic valve with ascending aortic dilation	BAVdil
endothelial microparticles	EMPs
C-reactive protein	CRP
apolipoprotein A1	ApoA1
apolipoprotein B100	ApoB100
